# Reward-enhanced encoding improves relearning of forgotten associations

**DOI:** 10.1038/s41598-018-26929-w

**Published:** 2018-06-04

**Authors:** Ewa A. Miendlarzewska, Sara Ciucci, Carlo V. Cannistraci, Daphne Bavelier, Sophie Schwartz

**Affiliations:** 1 Department of Neuroscience, University of Geneva, Geneva, Switzerland; 2 Swiss Center for Affective Sciences, University of Geneva, Geneva, Switzerland; 3 Geneva Finance Research Institute, University of Geneva, Geneva, Switzerland; 4 Geneva Neuroscience Center, University of Geneva, Geneva, Switzerland; 5 Psychology Section, FPSE, University of Geneva, Geneva, Switzerland; 6 Brain & Cognitive Sciences, University of Rochester, Rochester, NY, United States; 7 Biomedical Cybernetics Group, Biotechnology Center (BIOTEC), Center for Molecular and Cellular Bioengineering (CMCB), Center for Systems Biology Dresden (CSBD), Department of Physics, Technische Universität Dresden, Tatzberg 47/49, 01307 Dresden, Germany; 8 Brain Bio-Inspired Computing (BBC) Lab, IRCCS Centro Neurolesi “Bonino Pulejo”, Messina, 98124 Italy; 9 Lipotype GmbH, Tatzberg 47, 01307 Dresden, Germany

## Abstract

Research on human memory has shown that monetary incentives can enhance hippocampal memory consolidation and thereby protect memory traces from forgetting. However, it is not known whether initial reward may facilitate the recovery of already forgotten memories weeks after learning. Here, we investigated the influence of monetary reward on later relearning. Nineteen healthy human participants learned object-location associations, for half of which we offered money. Six weeks later, most of these associations had been forgotten as measured by a test of declarative memory. Yet, relearning in the absence of any reward was faster for the originally rewarded associations. Thus, associative memories encoded in a state of monetary reward motivation may persist in a latent form despite the failure to retrieve them explicitly. Alternatively, such facilitation could be analogous to the renewal effect observed in animal conditioning, whereby a reward-associated cue can reinstate anticipatory arousal, which would in turn modulate relearning. This finding has important implications for learning and education, suggesting that even when learned information is no longer accessible via explicit retrieval, the enduring effects of a past prospect of reward could facilitate its recovery.

## Introduction

Reward can enhance hippocampal memory consolidation and thereby protect memory traces from forgetting^1–3^. The positive influence of reward on memory may spread to associatively related stimuli^4^, and even to unpractised items from a rewarded category^5^. Recollection of an episode is a conscious process which involves the retrieval of information from memory, prompted by a critical cue, along with contextual details (e.g., remembering where or when a picture had been seen before^6^) and an accompanying subjective sense of recollection^7^. Pattern separation and pattern completion have been identified as key processes enabling recollection^8^. Pattern separation refers to the ability of the associative network to reduce the overlap between similar input patterns before they are stored in order to reduce the probability of interference during memory recall. Pattern completion refers to the ability of the network to retrieve stored patterns when presented with partial or degraded input patterns. Recent evidence for such operations being performed within the hippocampal structures has been provided by Neunuebel and Knierim (2014)^9^.

A recollection attempt may, however, result in failure to retrieve – a phenomenon called *forgetting*. Importantly, forgetting in declarative memory does not necessarily mean that a memory is lost. Rather, forgetting may result from impaired accessibility or lower coherence of a memory trace. For example, a memory trace may be easily retrievable immediately after learning, but at a later time point the aid of a specific cue may be necessary to recover it, due to its reduced accessibility. Thus, whereas forgetting may be defined as ‘the inability to recall something now that could be recalled on an earlier occasion’^10^, in this view of memory, forgetting may in fact be an inability to recall something now that could possibly be recalled on a later occasion^7^, perhaps when a critical cue or context is present. Evidence for such latent memory traces is well known in research on reward and fear conditioning: spontaneous recovery, reinstatement and rapid reacquisition are all phenomena that can occur after extinction of the original association. Similarly, in declarative memory, ‘savings’ – a reduction in the number of study trials or time required for relearning^11^ – have been observed for declaratively irretrievable associations. A memory may thus persist in a covert, inaccessible state after being forgotten, providing a platform for savings in future relearning.

**Figure 1:**
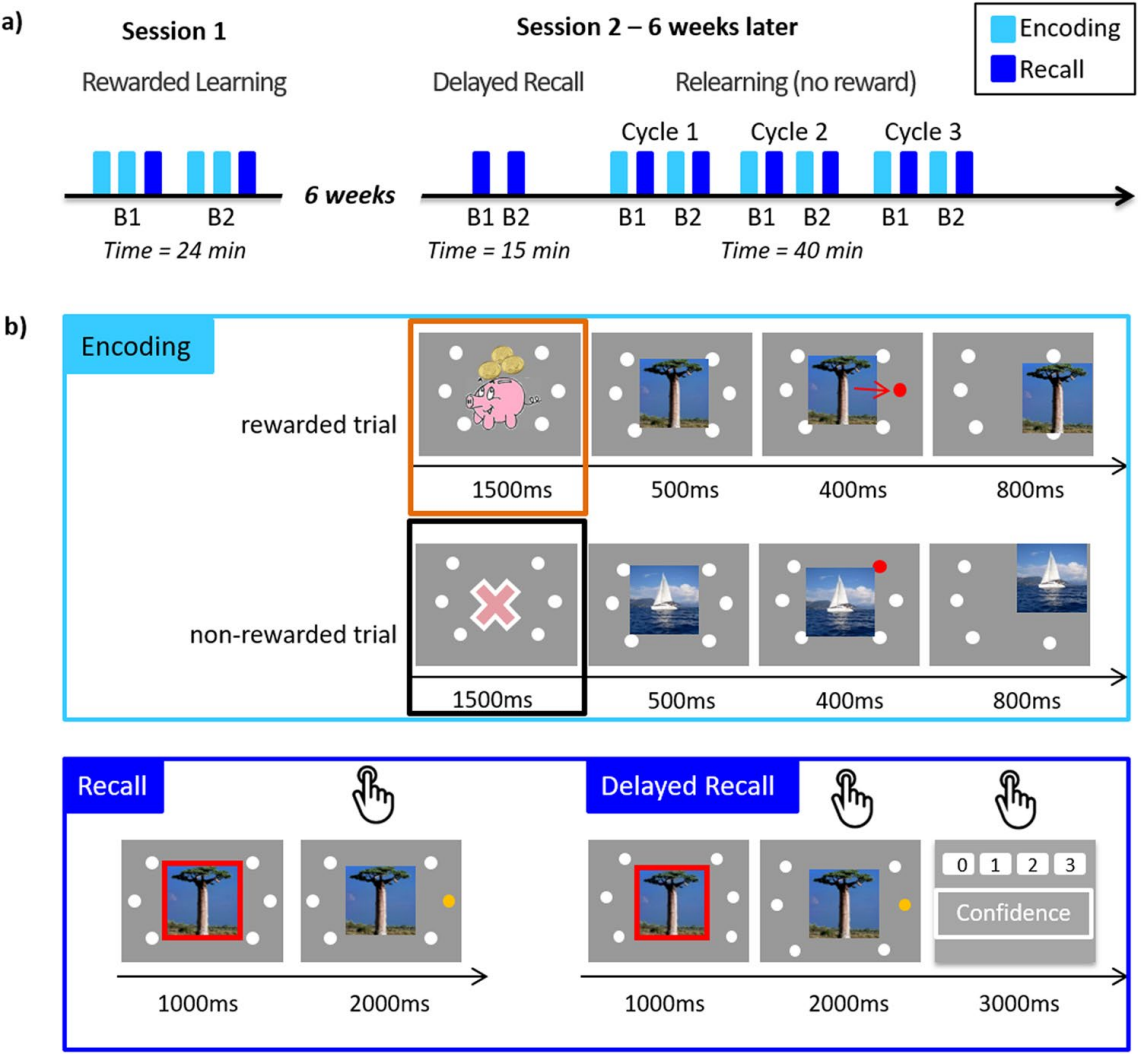
Experimental design (**a**) The experiment comprised two sessions separated by six weeks. During Session 1, participants encoded half of the rewarded and non-rewarded picture-location associations twice before being asked to recall them (followed by the recall block 1; B1). The same procedure was repeated for the second half of the associations (followed by the recall block 2; B2). Participants came back six weeks later to participate in Session 2. This session started with the same recall tasks as in Session 1. This was followed by a relearning task comprising 3 cycles of one encoding and one recall block. Importantly, relearning was administered in the absence of reward. (**b**) Encoding and recall. Encoding began with the presentation of a reward cue (a piggy with coins for the rewarded associations or a pink cross of the same size for the non-rewarded associations). Next, an image appeared centrally and moved towards one of the six locations of the screen. The participants’ task was to memorize the position corresponding to each picture Note that in the initial learning, reward was offered for pictures of one semantic category, which alternated every 9 trials (mini-blocks). Only at delayed recall, participants indicated their response confidence in addition to the remembered picture location. The piggy bank image was modified from http://coloringhome.com/coloring-page/1847657. Sailboat image was adapted from http://maxpixel.freegreatpicture.com/Adriatic-Sea-Sailboat-Summer-Boka-Boat-1824463 and is under CC0C Public Domain license. The boabab image is adapted from https://en.wikipedia.org/wiki/Adansonia_grandidieri#/media/File:Adansonia_grandidieri04.jpg licensed under CC-BY-SA.

While there is ample human research showing that reward benefits memory encoding^4,12,13^ and that rewarded (compared to non-rewarded) memory traces decay more slowly^2,14^, the question remains whether memories initially remembered better due to reward still benefit from a privileged status after they have been forgotten. In the present study, we thus focused on what happens after associations learned in anticipation of high reward motivation cannot be remembered anymore. To do so, we studied relearning after forgetting in participants who showed a reward-related memory enhancement. Accordingly, we selected participants who showed higher recall accuracy for associations encoded in anticipation of reward than for those encoded in anticipation of no reward. We hypothesized that, upon relearning, savings for memory traces which benefitted from reward will be larger than for non-rewarded ones. To test this hypothesis, we used a declarative memory task that relies on the hippocampus^15^, namely the cued explicit recall of learned object-location associations. Participants were tested immediately after the first learning during which half of the associations were rewarded, as well as after a delay of six weeks. In a key manipulation, those participants who showed a reward-memory advantage, were then asked to relearn exactly the same picture-location associations in the absence of reward in three repeated encoding-recall cycles (see Fig. 1 and Methods).

## Results

### Rewarded learning

**Figure 2:**
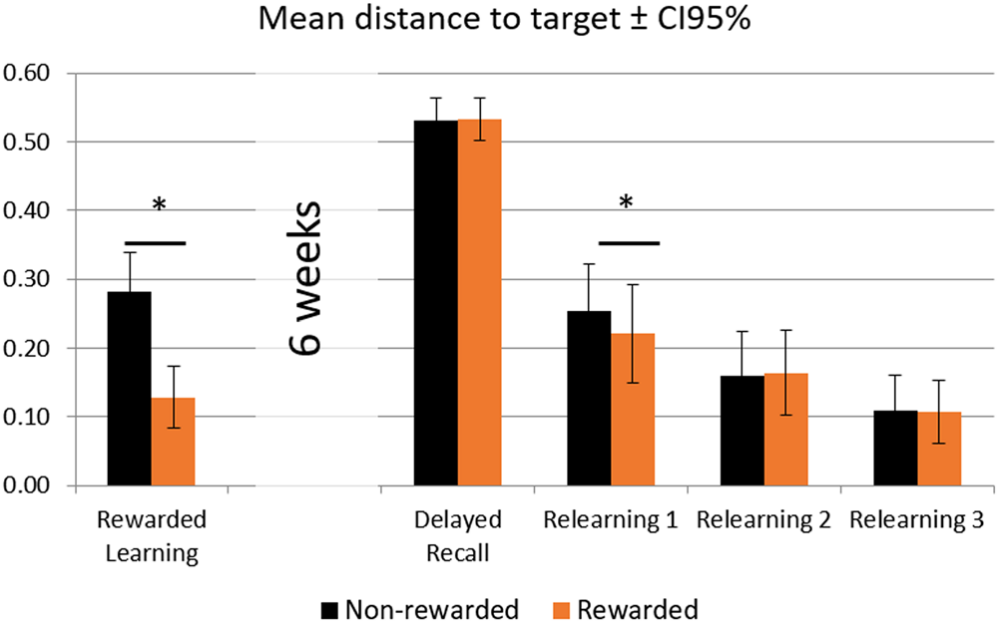
Mean distance to target (DTT). At learning, rewarded picture-location associations were better recalled (smaller DTT) than non-rewarded associations. This effect was abolished six weeks later at delayed recall. Yet, the effect of initial reward re-emerged during the first cycle of relearning of associations recalled incorrectly. Note that ‘rewarded’ and ‘non-rewarded’ labels after the six weeks refer to the reward status of the associations at rewarded learning (see Methods and Fig. 1a for details).

During the rewarded learning (Session 1), recall accuracy was comfortably above chance level (65.2% versus 16.67% for chance level) and showed an advantage for rewarded associations [mean % (SD): rewarded M = 75.58 (15.80), non-rewarded M = 55.4 (20.33); Wilcoxon sign-rank z = −3.824, p = 0.00001]. For every trial, we also calculated the Euclidean distance between the selected and studied location (one of six fixed locations on the screen; Fig. 1b), referred to as Distance-To-Target (DTT). DTT data were entered in a linear mixed model with factor Reward (rewarded, non-rewarded) and a random factor Subject. As expected, the DTT was significantly smaller for rewarded associations [F(1,1322.065) = 83.37, p-value < 0.001, *R*^2^_marginal_ = 0.054 and *R*^2^_conditional_ = 0.13; Fig. 2].

Statistical analyses performed on the reaction times (RTs) confirmed a main effect of reward [F(1,1322.025) = 4.575, p = 0.033, *R*^2^_marginal_ = 0.002, *R*^2^_conditional_ = 0.29, with faster responses on rewarded trials [mean ms (SD) rewarded: M = 1451.02 ms (451.67); non-rewarded: M = 1496.67 ms (460.08)]. Since it is reasonable to expect faster RTs on correctly recalled associations, we added a fixed factor Correctness (on target, off target), and found that there was indeed a main effect of Correctness [F(1,1328) = 85, p < 0.001, *R*^2^_marginal_ = 0.048 and *R*^2^_conditional_ = 0.344]. This analysis also revealed an interaction between Correctness and Reward due to incorrect responses being especially slow for rewarded associations [interaction Correctness × Reward, F(1,1322) = 4.13, p = 0.038, *η*^p^_2_ = 0.003]. In sum, the expected reward effects were all present at initial learning.

### Delayed recall

At delayed recall six weeks after the learning, accuracy for picture locations was at 21.2%. This is slightly above the chance level of 16.67% [sign-rank test against chance level z-value = 2.0975, p = 0.036]. Therefore, most of the follow-up analyses will include only those associations that were incorrectly recalled at this first delayed recall test administered at the very beginning of Session 2, and which we thereafter refer to as forgotten associations. Importantly, at this six week delayed recall, initially rewarded associations were not recalled better than non-rewarded ones [mean % (SD): M(rewarded) = 21.35 (9.46), M(non-rewarded) = 21.05 (6.95); Wilcoxon sign-rank z = 0.524, p = 0.68].

We also verified that there was no difference in the percentage of forgotten associations at delayed recall between rewarded and non-rewarded trials by performing a separate analysis considering only those associations that were initially correctly recalled during Session 1 [percentage of forgotten trials mean % (SD): M(rewarded) = 75.44 (10.1); M(non-rewarded) = 73.3 (12.1); sign-rank z = −0.8928, p = 0.372]. The analysis performed on DTT data further confirmed that six weeks after learning, initially rewarded associations were no longer more accurately recalled than non-rewarded ones [only incorrect responses: F(1,1034) = 0.058, p = 0.809]. Finally, RTs at delayed recall when considering only incorrect responses (i.e. those of interest for subsequent relearning), did not differ between rewarded and non-rewarded associations [main effect of Reward F(1,1016.474) = 2.268, p = 0.132].

At delayed recall, participants indicated confidence of their response on a scale from 0 to 3, where 0 – guessing, 1 – somewhat sure, 2 – quite sure, 3 – certain. When considering forgotten associations, there was no effect of reward on confidence ratings [F(1,1023) = 0.854, p = 0.356; M(rewarded incorrect) = −0.0497 (0.943), M(non-rewarded incorrect) = −0.1047 (0.96)].

We then asked whether response confidence had any effect on DTT, again taking into consideration initial reward status. The dependent measure DTT (z-scored) was entered in a linear mixed model with fixed factor Reward and with a trial-level covariate Confidence. For forgotten associations, neither confidence [F(1,1021) = 0.193, p = 0.663] nor reward [F(1,1021) = 0.057, p = 0.811] had an effect on DTT. Thus, six week after initial learning, forgotten associations showed no sensitivity to their initial reward status.

### Relearning

Relearning consisted of three cycles of re-encoding and recall (referred to as Relearning 1, 2, 3; see Fig. 1a), during which participants encoded the same picture-location associations as in Session 1, but without the prospect of any reward, and had then to recall the correct locations. We first checked that as expected performance improved as relearning progressed. This was indeed the case as shown by a main effect of relearning cycle in analyses focusing respectively on accuracy, DTT, and RTs for pictures recalled incorrectly at delayed recall [main effect of cycle: for accuracy F(2,90) = 42.157, p < 0.001, *R*^2^_marginal_ = 0.13 and *R*^2^_conditional_ = 0.84; for DTT F(2, 3015.978) = 54.44, p < 0.001, *R*^2^_marginal_ = 0.0308 and *R*^2^_conditional_ = 0.178; for RTs F(2,3016.069) = 4.541, p = 0.011, *R*^2^_marginal_ = 0.003 and *R*^2^_conditional_ = 0.205]. Given our interest in the role of previous reward in relearning, we first examined the first cycle of relearning where we expected possible effects of reinstated reward motivation to be the strongest. Indeed, the reactivation of prior reward associations could reinstate a state of reward motivation but it is likely to extinguish as the non-rewarded relearning progresses^16–18^. To assess relearning, we exclusively analysed forgotten associations, i.e. those associations incorrectly recalled at delayed recall. The analyses below include reward status at initial learning as a factor and have been carried out respectively on accuracy, DTT, and RTs as independent variables.

After the first relearning cycle, analysis of accuracy for forgotten associations showed a weak effect of reward history, whereby originally rewarded associations were relearned with a higher accuracy than non-rewarded associations [main effect of reward: z = −2.202, p = 0.028, paired samples sign-rank test]. A similar analysis on DTT indicated a main effect of reward [F(1,991) = 3.886, p = 0.049, *R*^2^_marginal_ = 0.003 and *R*^2^_conditional_ = 0.165], with originally rewarded associations being also relearned with smaller DTT (Fig. 2). These results establish that savings for initially rewarded associations that benefitted from immediate memory advantage may selectively enhance relearning six weeks later despite the fact that there was no difference at the delayed recall between rewarded and non-rewarded picture-location associations.

We found no effect of reward in later relearning cycles using the same linear mixed model analysis [main effect of reward in DTT in Relearning 2: accuracy z = −0.1184, p = 0.906, DTT F(1,996.241) = 0.001, p = 0.976; Relearning 3: accuracy z = 0.355, p = 0.722, DTT F(1,996.767) = 0.061, p = 0.804]. This effect is in accord with a possible extinction due to the absence of reward during relearning. A similar analysis was performed on RTs at relearning (for forgotten picture-location associations) with factors Relearning cycle (R1, R2, R3), Reward (rewarded, not rewarded at initial learning) and Correctness (on target, off target). We found only a main effect of correctness in R1 [F(1,1019.269) = 60.45, p < 0.001, *R*^2^_marginal_ = 0.053 and *R*^2^_conditional_ = 0.274] revealing faster RTs for correctly recalled associations [no effect of Reward, p = 0.55; no interaction Correctness × Reward, p = 0.436], as well as in R2 [Correctness F(1,1023.647) = 99.75, p < 0.001, *R*^2^_marginal_ = 0.08 and *R*^2^_conditional_ = 0.303; Reward p = 0.64; interaction p = 0.943] and in R3 [Correctness F(1,1020.462) = 70.01, p < 0.001, *R*^2^_marginal_ = 0.056 and *R*^2^_conditional_ = 0.307; Reward p = 0.384; interaction p = 0.975].

### Data-driven modelling

To further investigate the robustness of a role of reward at relearning, we turned to a multidimensional data-driven approach to complement the classical, hypothesis-driven statistical methods we have used so far. We performed a data-driven unsupervised multivariate analysis^19,20^ on the dataset from the delayed test and relearning cycles. Following the same logic as in the hypothesis-driven analysis, only forgotten associations were included in that analysis.

We considered DTT and RTs together as features to discriminate and search for patterns across the conditions in an unsupervised manner. In other words, we created a unique dataset that included for features the RT and DTT measures of individual trials for all 19 participants and for samples the 8 conditions: 2 reward status (initially rewarded or not) for the 4 memory tests (delayed recall, R1, R2, R3). We tested for the presence of any consistent variability across all these 8 conditions administered 6 weeks after the initial learning. We applied an unsupervised (meaning that the algorithm did not use any information about the reward status and recall cycle of each of the 8 conditions) machine learning procedure allowing dimensionality reduction by extracting groups of common features across the whole dataset and compressing them in a reduced space along diverse dimensions of embedding. This new reduced space could thus represent and emphasize differences between the conditions that account for a major source of variability. For instance, if the reward significantly influenced the 8 conditions represented by the multidimensional combination of DTT and RTs, we would expect to find that the unsupervised analysis is able to compress in the reduced space of visualization a pattern of condition-segregation that matches with the original reward status. We compared two parameter-free algorithms, which therefore do not require the tuning of any internal parameter and avoid data overfitting: one (principal component analysis, PCA^21^) using a linear and one (minimum curvilinear embedding, MCE^22,23^) a nonlinear dimension reduction transformation. The latter algorithm allows the analysis of all behavioural data at the same time without the constraints of linearity present in all classical statistical approaches we have used so far. Further explanations concerning the choice of these specific algorithms are provided in the Methods section.

**Figure 3:**
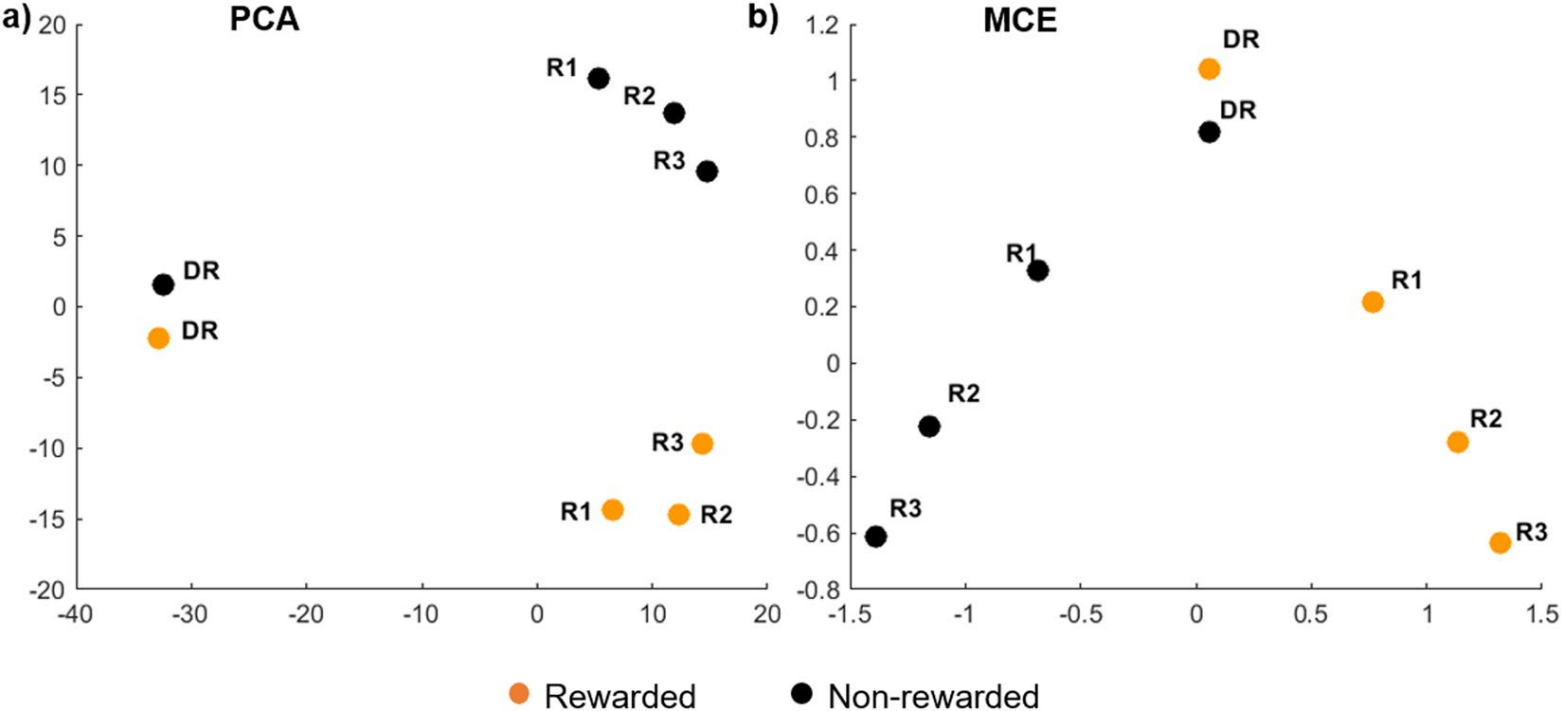
Unsupervised (data-driven) dimension reduction pattern recognition analyses performed on dataset of Session 2 recall tests for pictures forgotten at delayed recall. Conditions previously associated with reward are displayed with orange dots, whereas conditions previously associated with no reward are displayed with black dots. The abbreviations next to the dots mean: DR for delayed recall; R1, R2, R3 for relearning cycle 1,2,3. (**a**) 1st dimension of the principal component analysis (PCA) provides evidence for the effects of learning on performance, while the 2^nd^ dimension shows a trend for the effect of reward. (**b**) Minimum curvilinear embedding (MCE) shows a significant and neat difference on the 1^st^ dimension due to previous reward association. On the other hand, the 2^nd^ dimension of MCE provides an ordering that perfectly matches with the relearning cycles.

The results of the analysis using PCA and MCE are displayed in Fig. 3 and both confirm a clear and eye-catching data separation for type of recall test and for reward status. On the left (Fig. 3a), PCA compresses on the first dimension a discriminative variability that accounts for the type of recall test. In particular, the delayed-recall test is markedly separated from the relearning ones. PCA’s second dimension separates originally rewarded trials (scoring low on the PC2) from non-rewarded ones (scoring high on PC2). In our dataset, the first two principal components of the PCA explain more than half (55%) of the variance in the data (39.99% for PC1 and 14.61% for PC2). The explained variance for PC3 is 12.48% while its value for PC4 is 10.37%. These results are further confirmed by MCE (Fig. 3b) that shows an even clearer separation for type of recall test and for reward status. The first dimension of MCE compresses a discriminative variability that offers a symmetric separation of the conditions related with reward, with the originally rewarded conditions scoring high and non-rewarded ones scoring low on this first dimension. Interestingly, the second dimension of MCE displays a rather ordered and progressive separation of the relearning recall tests (Fig. 3b). Note that in general, both for PCA and MCE, the distance due to reward was notably smaller for delayed recall than for relearning, consistent with the results from our hypothesis-driven analyses.

## Discussion

Forgetting has been proposed as a key process in memory formation^24^ and as such, it is affected by the reward-triggered dopaminergic modulation. Yet, although it has been shown that rewarded memories are forgotten more slowly^25^, it has not been investigated whether persistent effects of monetary reward on initial memory can still affect subsequently forgotten (declaratively inaccessible) memory traces. Our results show that offering monetary reward at encoding may facilitate not only retention but also relearning^26^ of forgotten hippocampus-dependent memories.

We compared the fate of previously rewarded (and better remembered) and non-rewarded object-location associations after they had been forgotten due to passage of time in 19 healthy participants, and tested whether reward during the initial learning would still modulate the relearning of the same associations 6 weeks later. During learning, reward improved memory performance (reducing distance to target and increasing accuracy), replicating the known effect of reward on initial acquisition^2^. Six weeks later, explicit recall performance for initially rewarded and non-rewarded associations did not differ as indicated by comparable accuracy, DTT, and RTs. Critically, no effect of reward status at initial learning was observed when considering forgotten associations, namely those for which we then investigated relearning. As hypothesized, subsequent relearning of forgotten associations was faster for initially rewarded associations for which we found a memory advantage compared to non-rewarded ones. This effect was observed in the absence of monetary reward and was most marked early during relearning, suggesting early spontaneous recovery of a reward response^27^.

The savings in relearning cycles 2 and 3 were comparatively reduced, which could be attributed to extinction of reward response since cycles 1, 2 and 3 each entailed the presentation of all pictures and their positions in the encoding phase followed by the recall phase in the absence of any reward (unlike initial learning in session 1 six weeks before). Previous human studies have shown that extinction of appetitive reward response in a conditioning task with monetary reward occurs already after the first 20 trials^28^ and therefore, we suspect that a similar effect took place in our memory task with previously reward-associated stimuli. As a consequence of extinction of reward motivation, we would no longer observe any differential effects due to reward status during cycle 2 and 3 of relearning.

A second possible explanation for the lack of reward-related savings relearning 2 and 3 is that such effects may be overshadowed by a ceiling effect in memory performance. Please note that the participants effectively relearned all associations in the first relearning cycle going from accuracy of 0 to >60% (data presented in the Table 1 below). Subsequent additional learning proceeded much slower, with accuracy reaching 75% in the second cycle (∆ of ~12%) and an increase of only ~7.5% between cycles 2 and 3 (to ~83%).

**Table 1:** Memory accuracy for associations forgotten at delayed test reported as mean ± st. deviation.

	Relearning 1	Relearning 2	Relearning 3
Rewarded	64.9% (23%)	74.8% (21%)	82.7% (17%)
Non-rewarded	61.5% (23%)	75.6% (20%)	82.6% (17%)

The presence of an effect of reward in relearning was confirmed using a data-driven multivariate approach combining both DTT and RTs from the non-rewarded second session of the experiment. We demonstrated that the reward effect was detected with both a linear (PCA) and a nonlinear unsupervised algorithm (MCE), confirming and extending our classical statistical analyses. This result additionally illustrates the potential of using data-driven methods for behavioural data analysis, especially in datasets with significant inter-individual differences where nonlinear patterns can be expected.

Our findings of a lasting influence of reward memory on subsequent mnemonic processes extend the known role of reward on memory formation beyond encoding, early consolidation (reviewed in^4^), and late consolidation^29,30^ stages of memory. Moreover, they are in line with previous observations of enduring effects of reward on, for instance, choice and preferences^31^. Lingering effects of reward on memory may be mediated by the anatomical and functional connections between the hippocampus, the dopaminergic midbrain and the ventral striatum, which form the hippocampal-VTA loop that supports motivated memory formation^32^. Future studies using neuroimaging (fMRI) could examine the possible reactivation of reward areas (e.g. VTA, ventral striatum) during delayed recall and relearning. We suggest that the present behavioural results could implicate either a reactivation of the reward system, signalling the re-emergence of the original reward memory^33^, or increased hippocampal-VTA functional connectivity, signalling a strengthening (reconsolidation) of the original memory^34^ at relearning.

The phenomenon of memory recovering following forgetting bears some analogies with an effect known as ‘renewal’ that occurs after the extinction of a conditioned response in a particular context. Renewal is a special case of the return of a memory that happens without additional exposure to the unconditional stimulus (a reward), in contrast to two other phenomena - reinstatement and rapid reacquisition. Studies of appetitive conditioning in animals explain the accelerated return of a conditioned response after its successful extinction as arousal-mediated^35^. Upon re-exposure to the reward-conditioned stimulus (CS+), re-learning is enhanced by the reactivation of the unconditional stimulus memory that elicits arousal, possibly due to the re-evoked state of reward anticipation. Conversely, relearning is slower when the CS+ is not presented. Therefore, the lasting influence of reward that was revealed in savings at relearning in our spatial memory task could be explained by the reactivation of associated reward memories, which in turn may reinstate motivation and promote re-encoding and reconsolidation. This last finding – of a latent effect of reward motivation – may have important implications for learning and education^36–38^.

## Methods

### Participants

Thirty-four healthy volunteers recruited at the University of Geneva participated in the study. Since the main focus of the study was to examine the memory performance of previously reward-enhanced memories during subsequent relearning, only participants who showed a reward-related advantage at learning were invited for Session 2 (Fig. 1a). To determine the reward-related advantage in memory, we compared the mean distance to target (DTT) for all rewarded and all non-rewarded trials at first recall for each participant. Participants whose mean DTT was equal or larger for rewarded compared to non-rewarded trials (n = 11) received a financial compensation for their time and were excluded from analysis. Three further participants were excluded from analyses: one due to technical issues, one due to awareness of the semantic category-reward manipulation, and one due to above-average memory performance at delayed recall (2 standard deviations above group mean). Nineteen participants were thus included in the analyses (10 females; mean age ± SD: 25.7 ± 5.05). All participants were students or recent graduates with no declared history of neurological or psychiatric disease and no sleep problems. All participants gave written informed consent. The study protocol was approved by the Ethics Committee of the Geneva University Hospitals and was performed in accordance with relevant guidelines and regulations.

### Stimuli

We used as stimuli seventy-two unique natural photographs portraying activities, scenes, animals and vehicles belonging to two semantic categories (36 pictures from each), “sea” and “savanna”. The photographs were trimmed to measure 512 × 512 pixels and were presented on a screen of 1280 × 1024 resolution (screen size 47 × 57 cm) at a distance of ~60 cm. The stimuli thus subtended 22 × 22 degrees of visual angle. The two sets of pictures, corresponding to the two categories, were selected from a large picture dataset (n = 150) based on ratings performed by a group of 10 independent raters such that they were neutral in valence and did not differ in terms of arousal, familiarity, and how interesting their content and visual composition was. The two sets did not differ in terms of spatial frequencies, and mean luminance was equalized over the sets.

### Procedure

The experiment was composed of two sessions scheduled six weeks (±3 days) apart and was performed on a desktop computer. Session 1 consisted of an initial learning task in which participants encoded the location (among 6 possible locations) of each of the 72 pictures. During Session 2, participants first performed a delayed recall test (DR), and then proceeded to the relearning task, which was composed of 3 encoding-recall cycles (R1, R2, R3; Fig. 1a). The tasks were programmed and presented using Cogent toolbox (Cogent 2000, v.1.32, http://www.vislab.ucl.ac.uk/cogent_2000) implemented in Matlab v7.9 (R2009b, The MathWorks, Inc., Natick, Massachusetts, United States).

For the initial learning, the 72 different pictures were presented in 2 blocks of 36 pictures each. Each block was composed of 4 mini-blocks of 9 pictures, i.e. 2 for each semantic category (“the sea” and “savanna”). Assignment of reward to one of the categories was counterbalanced across participants. Each block was therefore composed of 2 mini-blocks of rewarded and non-rewarded pictures presented in a pseudo-randomized order (RNRN or NRNR).

During Session 1 (learning), each of the two blocks of different 36 pictures was presented twice in two successive runs of encoding followed by one run of recall (Fig. 1a top). The runs were separated by 10 s, while block 1 and 2 were separated by a pause of 60 s. During encoding, each mini-block started with a reward or no-reward cue presented for 1.5 s. The reward cue was a pink piggy-bank with animated coins. The non-reward cue was a cross of the same colour and size. In later trials of the mini-block, a scaled-down cue was presented before each picture as a reminder (Fig. 1b). No reward cue was presented during recall. At each encoding/recall run, the order of pictures within the mini-block as well as the order of mini-blocks changed.

Participants were asked to memorize the location of pictures on the screen during the encoding runs and were told that for trials in a mini-block starting with the reward cue – their correct response in a later recall run would be rewarded with bonus points (Fig. 1b). A maximum of 10 Swiss francs was offered for their performance (for indicating the correct screen position of all 36 rewarded pictures; that is 27c per one picture) in addition to the regular hourly compensation of 15 Swiss francs. Participants were instructed about the encoding-recall structure, and were warned not to rely on the temporal sequence of the trials as it changed across the encoding and recall blocks. A practice run was administered before the experiment with a separate set of 18 black-and-white drawings.

In the recall run (Fig. 1b bottom), participants pressed one of 6 coloured keyboard keys corresponding to the chosen location using both hands (3 keys per hand). Participants were encouraged to respond on every trial, even if unsure of their choice. They were instructed to withhold their response until 1 s after image onset (indicated with a red frame) but also early responses were included in analysis. No feedback was provided during recall and participants saw their performance and the monetary reward only at the very end of the Session 1.

Only one participant was aware of the reward-semantic category assignment and her data have been excluded from subsequent analyses. Participants were scheduled to return six weeks later for Session 2 which they were told would be a continuation of the experiment but no information was provided about the follow-up task. At final debriefing at the very end of Session 2, we learned that none of the participants had expected a memory test.

Six weeks later, during Session 2, participants were asked to recall the location of all 72 pictures learned during Session 1 and to state the confidence of their response on a 4-point scale (3 = ‘certain’, 2 = ‘rather sure’, 1 = ‘somewhat sure’, 0 = ‘guessing’; bottom right in Fig. 1b). The recall test maintained the mini-block structure used during initial learning, and the pictures were again shuffled within each mini-block. Following this delayed recall task, participants started relearning, which consisted of three cycles of single encoding-recall runs (see schema on the right in Fig. 1a). Exactly the same picture-location associations were used as for the initial learning of Session 1. The task was similar to the learning one but differed by two main aspects: there was only one encoding run followed by recall after every 36 trials, and no reward was offered and thus no reward cues were presented. No feedback was provided at any point in the relearning task.

### Data Analysis

For each condition and cycle, memory performance was measured as accuracy

1 $$(\frac{\#correct\,responses}{\#trials\,per\,condition}\times 100),$$

as well as distance to target (DTT). Euclidean distance to target was calculated as

2 $$\sqrt{{({x}_{n}-{x}_{0})}^{2}+\,{({y}_{n}-{y}_{0})}^{2}}$$

where n is the participant’s response and 0 is the target position on a 1280 × 1024 pixel monitor (see Fig. 1b). Consequently, DTT measures could take 6 possible values, including 0 for a correct response. For analyses, we used scaled DTTs obtained by dividing the Euclidean distance values by the maximum possible value (1068.5). In the hypothesis-driven analyses non-responses were excluded and reaction time (RT) data were logarithm(10)-transformed. For both RT and DTT, the first response was considered, unless the RT was <100 ms in which case it was regarded as impulsive and excluded from analysis. Confidence of response data (categorical values 0–3) were normalized within each participant (z-score).

We used two types of statistical analyses: univariate hypothesis-driven and multidimensional data-driven (unsupervised). In order to capture the individual differences that usually characterize memory tasks and retain the information related with response variability that is lost with data reduction due to averaging, behavioural data for the recall of picture locations were first analysed at a single trial level using a linear mixed model^39^ (which is univariate and hypothesis-driven) implemented in SPSS v.22 (IBM Corp. Released 2013. IBM SPSS Statistics for Windows, Armonk, NY: IBM Corp.). A linear mixed model (random effects model) accounts for within-subject correlation of repeated measurements with the inclusion of a random intercept for subjects. Note that linear mixed model analyses do not require the data to be normally distributed. In addition, mixed models can handle unbalanced data (e.g., unequal trials per condition) and covariates that vary continuously with every data point, like response confidence that varied at the trial-level in Session 2. When used together in a model (one as a dependent variable and one as a covariate), DTT and confidence scores were standardized (z-scoring normalization) for the optimization of the mixed linear model estimation. The purpose of standardization and scaling of the covariates was to make the values more similar to the dependent variable in the model. Random intercept was included in the reported model when the Wald Z value was significant, indicating that a significant proportion of residual variance is due to repeated measures (trials) in subjects.

In the analysis of accuracy, computed as percentage of correct (on-target) responses relative to all trials, we used nonparametric tests (Wilcoxon sign-rank) to test for a difference between responses from initially rewarded and non-rewarded trials because the distribution of the data was not Gaussian. We present group means with confidence intervals (95%) in Fig. 2.

For effects statistically significant at α < 0.05, we report effect sizes in the form of the coefficient of determination *R*^2^ for the reported fixed effect as well as for the fixed effect + random effect of random intercept (factor Subject). It is defined as the proportion of variance in the response variable that is explained by the explanatory variables. We report both *marginal* and *conditional R*^2^, that gauge, respectively, the contributions of fixed, and of fixed *and* random effects of variation in the responses^40^.

To further explore the possible latent influence of reward on performance during relearning, DTT and RTs from the associations forgotten in Session 2 were analysed with a multidimensional data-driven approach. We chose to include only that part of the experiment where no actual reward was offered in order to focus solely on our question of interest, which is the lingering effects of initial reward when relearning in the absence of reward. The aim of this analysis was to unsupervisedly detect and distinguish the factors that influence the relations between the experimental conditions. We performed a linear and nonlinear multivariate analysis (dimensionality reduction) by means of two parameter-free unsupervised machine learning algorithms: principal components analysis (PCA) for the linear analysis and Minimum Curvilinear Embedding (MCE) for the nonlinear analysis. We chose PCA because it is the mainstream linear multivariate method to unsupervisedly explore data patterns in multidimensional data^22^. For comparison, we chose MCE^22,23^ - a nonlinear version of PCA that is also parameter-free and that demonstrated to achieve top performance in unfolding patterns in many applications from biology and medicine to radar signal analysis^22,41–47^. MCE has in particular the advantage to be the only parameter-free nonlinear machine learning approach that was specifically designed to deal with small size datasets, as is the case in our study^22,23^.

The data were DTT and RTs for associations that were incorrectly recalled (i.e., forgotten associations) at the delayed recall at all of the recall tests in Session 2. As a result, this dataset has 8 conditions: delayed recall and three relearning recall tests for the originally rewarded and non-rewarded pictures. Here, both RTs and DTTs were zscored (i.e., mean-centred and scaled to have standard deviation 1.). Each of the 8 conditions constituted a row in the data matrix and was defined by 1040 features, which is an aggregate of a variable number of 21–33 trials for 19 subjects for RTs and DTTs. Therefore, the dataset matrix had 8 rows and 1040 columns. As noted in the hypothesis-driven analysis, we noted a strong effect of individual differences in performance that is often characteristic for memory tasks, including the current dataset. Since we z-scored each feature (column) of the dataset (representing one trial per participant), each feature is mean-centered, ensuring that the first component of PCA describes the direction of maximum variance^48^. Since an equal number of data points is required for this analysis, missing data were replaced with either the maximum of response time or distance to target that is in both cases 1.

For PCA, the data were factorized using singular value decomposition. The dataset was also analysed using MCE which is a form of parameter-free nonlinear-kernel PCA designed for nonlinear dimensionality reduction. We used a noncentered MCE that does not centre the minimum curvilinear kernel (hence the first dimension of embedding should be neglected, and we renamed the second dimension as first dimension, the third as second), because it was shown to be more effective especially when time-varying (time-dependent trajectory of the conditions in the multidimensional space) effects are influencing the conditions^23,43,45^. Sample labels (if known) are not used for the data projection thus rendering the analysis unsupervised. Using the MCE algorithm, we searched for the hidden pattern (specifically: the ordering of the conditions on one of the first two dimensions) that unsupervisedly emerge explaining the higher variability in the data without the PCA’s constraint to map only linear variability. To visually verify if any matching between represented condition-variability in the 2D reduced space and known condition-labels (recall tests: delayed recall, relearning tests R1, R2, R3; or reward/no-reward status at learning) was present, the condition points plotted in the 2D reduced space (for both PCA and MCE) are marked in a colour that represents the presence or absence of reward, and text labels report the recall cycle. The results of this analysis are displayed in Fig. 3. Since the patterns obtained by PCA and MCE are matching with a perfect separation there is no need to apply statistical or geometrical evaluations (such as class separability measures) in the 2D reduced space to quantify the level of separation.

### Data availability

The datasets generated during and/or analysed during the current study are available from the corresponding author on reasonable request.
